# Donor telomeres and their magnitude of shortening post-allogeneic haematopoietic cell transplant impact survival for patients with early-stage leukaemia or myelodysplastic syndrome

**DOI:** 10.1016/j.ebiom.2025.105641

**Published:** 2025-03-08

**Authors:** Shahinaz M. Gadalla, Hormuzd A. Katki, Tsung-Po Lai, Paul L. Auer, Casey L. Dagnall, Caitrin Bupp, Amy A. Hutchinson, James J. Anderson, Kyra J.W. Mendez, Stephen R. Spellman, Valerie Stewart, Sharon A. Savage, Stephanie J. Lee, John E. Levine, Wael Saber, Abraham Aviv

**Affiliations:** a Division of Cancer Epidemiology and Genetics, National Cancer Institute, Bethesda, MD, USA; b Center of Human Development and Aging, New Jersey Medical School, Rutgers, NJ, USA; c Division of Biostatistics, Institute for Health and Equity, and Cancer Center, Medical College of Wisconsin, Milwaukee, WI, USA; d Cancer Genomics Research Laboratory, Frederick National Laboratory for Cancer Research, Rockville, MD, USA; e Center for International Blood and Marrow Transplant Research, NMDP, Minneapolis, MN, USA; f College of the Environment, University of Washington, Seattle, WA, USA; g Center for International Blood and Marrow Transplant Research, Medical College of Wisconsin, Milwaukee, WI, USA; h Clinical Research Division, Fred Hutchinson Cancer Center, Seattle, WA, USA; i Tisch Cancer Institute, Icahn School of Medicine at Mount Sinai, New York, NY, USA; j Division of Hematology and Oncology, Medical College of Wisconsin, Milwaukee, WI, USA

**Keywords:** Telomeres, Telomere length, Haematopoietic cell transplant, Donor selection, Relapse

## Abstract

**Background:**

Donor selection is a key success factor in allogeneic haematopoietic cell transplant (HCT). We evaluated the potential impact of donor leucocyte telomere length (LTL) and LTL shortening in recipients at three-month post-HCT (LTL-3MS) on the two-year HCT outcomes.

**Methods:**

We identified a cohort of 384 HCT recipients for early-stage leukaemia or myelodysplastic syndrome in the Blood and Marrow Transplant Clinical Trial Network protocol#1202 with blood samples collected three-month post-HCT. Blood samples from respective donors were available at the Centre for International Blood and Marrow Transplant Research biorepository. We used Cox proportional hazards models for statistical analyses.

**Findings:**

A better two-year overall survival (OS) was associated with longer donor LTL (adjusted-hazard ratio [HR] = 0.60, 95% confidence interval [CI] = 0.37–0.96, for LTL ≥6.7 kb vs LTL< 6.7 kb, p = 0.03), and higher LTL-3MS (HR = 0.52, 95% CI = 0.34–0.80, for LTL-3MS ≥ 230 vs < 230 bp, p = 0.003). Longer donor LTL was associated with a lower risk of non-relapse mortality (NRM; HR = 0.48, p = 0.05), while higher LTL-3MS was associated with lower relapse risk (HR for relapse risk = 0.53, p = 0.008). The adjusted 2-year cumulative risk of all-cause mortality was reduced by about half for patients with both donor LTL ≥6.7 kb and LTL-3MS ≥ 230 bp vs patients with neither characteristic (21% vs 41%, respectively; p < 0.0001).

**Interpretation:**

Selection of donors with longer LTL may improve HCT outcomes. Limited LTL shortening in recipients post-HCT may guide relapse prediction.

**Funding:**

The NCI intramural research program and NIH grant U01AG066529.

**Research in context**

**Evidence before this study**

Longer donor leucocyte telomeres increase survival of recipients undergoing haematopoietic cell transplant (HCT) for severe aplastic anaemia, but this relation has not been established in HCT recipients for haematological malignancies. In addition, as donated haematopoietic cells reconstitute the recipient haematopoietic system, they experience extensive telomere shortening, but the relation between this shortening and recipient outcomes post-HCT is unclear.

**Added value of this study**

Using precise telomere length measurements by Southern blotting, this study shows that HCT recipients for early-stage leukaemia or myelodysplastic syndrome experienced improved two-year overall survival when (i) donor leucocyte telomeres were longer pre-HCT, and (ii) the shortening of leucocyte telomeres in the recipient was greater three-month post-HCT. More leucocyte telomere shortening in the recipients was associated with lower risk of relapse.

**Implications of all the available evidence**

Measurements of donor leucocyte telomere length will help selecting donors for better HCT recipient outcomes.

## Introduction

Recipients of allogeneic haematopoietic cell transplant (HCT) remain susceptible to high mortality and morbidity,^1^ which can be reduced by optimizing donor selection.^2^ Histocompatibility matching and young age are at present the only donor factors associated with improved recipient overall survival (OS) after HCT.^3^^,^^4^ The specific biological reasons behind the better survival among HCT recipients from young donors are not well understood. One contributing factor could be the longer telomeres of HCs donated by younger individuals.

Telomeres shorten with the replication of somatic cells, including HCs.^5^ Telomere length (TL) is highly variable among individuals starting from birth and naturally shortens with age.^6^^,^^7^ After HCT, donated HCs experience rapid telomere shortening because of their massive proliferation as they reconstitute the recipient's haematopoietic and immune systems.^8^^,^^9^ As critically short telomeres signal cessation of replication, donated HCs with short telomeres may have less replicative capacity,^10^ and thus negatively affect HCT outcomes. We thus hypothesized that TL is a better predictor for donated HC replicative capacity than age and that it would be associated with patient survival after HCT independently of donor age.

## Methods

### Patient and sample selection

HCT recipients were selected from the prospective multicentre cohort of the Blood and Marrow Transplant Clinical Trial Network (BMT CTN) Protocol 1202.^11^ The original protocol provided a biorepository resource with high-quality clinical data to evaluate predictive biomarkers of HCT complications and associated mortality. Patients were included in the current study if they: 1) received HCT for acute or chronic leukaemia or myelodysplastic syndromes (MDS); 2) at an early stage, defined as first complete remission for leukaemia. For MDS, the following criteria defined early-stage: refractory anaemia with ringed sideroblasts, refractory cytopenia with multilineage dysplasia, and blast count <5%, or MDS with isolated 5q-syndrome^12^; 3) had available blood sample collected at three months post-HCT; and 4) had an available blood sample from their respective donor at the Centre for International Blood and Marrow Transplant Research (CIBMTR) repository operated by the NMDP. Donor blood samples for TL measurements were collected before bone marrow aspiration or mobilization of peripheral blood grafts. All eligible patients were included in the study.

### Study end points and definitions

The study primary endpoint was the two-year OS post-HCT, defined as death from any cause. Secondary endpoints included: a) non-relapse mortality (NRM), defined as death during continuous complete remission following HCT, and b) relapse, defined as disease recurrence after reaching complete remission. Death after relapse was considered relapse mortality. Follow-up started at the date of post-HCT sample collection (mean ± standard deviation; SD = 90 ± 14 days) and ended at the outcome of interest or censored two years post-HCT.

### Telomere length measurements

TL in DNA extracted from peripheral blood samples was measured by two methods: Southern blotting of the terminal restriction fragments (SB) and quantitative polymerase chain reaction (qPCR).^13^, ^14^, ^15^ SB measurements (referred to as LTL) are expressed in base pairs (bp) or kilobases (kb, which = 1000 bp); qPCR measurements are expressed as the ratio of the telomeric PCR product to that of a single copy gene (referred to as LT/S). The intraclass correlations for the LTL and LT/S were 0.98 and 0.82, respectively. The study main analyses used LTL because SB is the gold standard TL assay that expresses data in absolute TL units. Because this research was also supported by the NIH-funded Telomere Research Network (TRN; https://trn.tulane.edu), which aims to evaluate in clinical settings whether qPCR measurements of LTL align with SB measurements in capturing the role of telomeres in human health, we also examined the association of LT/S with HCT outcomes.

### Statistical analysis

We used Kaplan–Meier and cumulative incidence estimators to calculate the probabilities of OS and the cumulative incidence for NRM and disease relapse. For multivariable analyses, we calculated the hazard ratios (HRs) and 95% confidence intervals (CIs) for OS, using Cox proportional hazard models, and cause-specific hazards for NRM and relapse incidence (as each was a competing risk to the other).

Clinical parameters (Table 1) were tested in a stepwise procedure for inclusion in the models with a p-value threshold of 0.15 for model entry and retention. Donor age was forced into all final models because of its possible confounding effect on the association between donor LTL and post-HCT outcomes. The proportional hazards assumption was tested for all variables based on Schoenfeld residuals test with no violation. We also tested for interactions between LTL parameters and clinical covariates; all were non-statistically significant. Final models for OS were adjusted for donor and recipient ages, recipient sex, disease type, and HCT comorbidity index (HCT-ci). NRM models were adjusted for recipient and donor age and HCT-ci. Relapse models were adjusted for recipient and donor age, HCT-ci, and donor type.

**Table 1:** Characteristics of study participants.

Variables	N (%) total = 384
**Recipient age (Years)**	
3–18	33 (8.59)
>18–35	50 (13.02)
>35–50	75 (19.53)
>50–65	148 (38.54)
>65	78 (20.31)
**Donor age (Years)**	
2–18	12 (3.13)
>18–35	187 (48.70)
>35–45	57 (14.84)
>45–55	54 (14.06)
>55	74 (19.27)
**Recipient race**	
White	330 (85.94)
African American	28 (7.29)
Others/unknown	26 (6.77)
**Recipient sex**	
Male	229 (59.64)
Female	155 (40.36)
**Donor sex**	
Male	252 (65.63)
Female	132 (34.38)
**Disease**	
AML	229 (59.64)
ALL	86 (22.40)
CLL	4 (1.04)
CML	11 (2.86)
MDS	54 (14.06)
**HCT-CI**	
High	171 (44.53)
Intermediate	128 (33.33)
Low	85 (22.14)
**Graft source**	
Bone marrow	83 (21.61)
Peripheral blood	301 (78.39)
**Karnofsky performance score**	
90–100	223 (58.07)
≤80	160 (41.67)
Unknown	1 (0.26)
**Donor type**	
Matched sibling	139 (36.20)
Unrelated matched	181 (47.14)
Unrelated mismatched	29 (7.55)
Haplo-identical	35 (9.11)
**Conditioning intensity**	
Myeloablative	243 (63.28)
Reduced intensity	118 (30.73)
Non-myeloablative	23 (5.99)
**GVHD prophylaxis**	
Post-transplant cyclophosphamide	27 (7.03)
Methotrexate-based (MTX)	236 (61.46)
Calcineurin-inhibitor based (no MTX)	98 (25.52)
Other^a^	23 (5.99)
**Year at HCT**	
2013–2014	278 (72.40)
2015–2016	106 (72.60)

Abbreviations: AML, acute myeloid leukaemia; ALL, acute lymphoblastic leukaemia; CML, chronic myeloid leukaemia; CLL, chronic lymphocytic leukaemia; MDS, myelodysplastic syndrome; CI, comorbidity index; GVHD, graft-vs-host disease.

a Includes 3 ex-vivo T cell depletion and 15 CD34 selection.

The LTL parameters included in the analyses, tested in separate models, were donor LTL and the magnitude of LTL shortening in the recipient at an average of three months post-HCT. LTL-three-month shortening (LTL-3MS) was calculated as the donor LTL minus that of the matched recipient three months post-HCT, expressed in bp.

To define the pattern and shape of associations with the primary end point, the relationship between OS and LTL parameters were tested on a continuous scale (Supplemental Table S1) and in quartile categories. The visual inspection of the survival curves suggested the use of the upper limit of the lowest quartile of TL parameters as cut-off thresholds (6.78 kb for donor LTL and 222 bp for LTL-3MS). For all analyses, we used 6.7 kb for donor LTL and 230 bp for LTL-3MS for easier clinical adaptation (Supplemental Figure S1). The distribution of LTL parameters is presented in Supplemental Table S2.

To better guide clinical decision-making, we calculated the 2-year absolute cumulative risk of mortality associated with mutually adjusted donor LTL and LTL-3MS by fitting cause-specific Cox models to mortality caused by relapse or non-relapse using the covariates used for the relative risk models. We estimated the monthly all-cause mortality cumulative risk for each person using their covariates by summing the estimated risk of mortality from relapse and non-relapse causes (each is considered a competing risk to the other). Then we averaged the calculated absolute cumulative risk and plotted them over the first two years after HCT within categories of TL parameters (donor LTL of <6.7 kb or ≥ 6.7 kb, and LTL-3MS of <230 bp or ≥ 230 bp). In the subset with matched donor transplants (matched sibling and 8/8 unrelated donors combined), we report the adjusted cumulative risk of 2-year mortality within categories of donor age (younger, i.e., ≤35 years, or older, i.e., >35 years), and donor LTL (shorter, i.e., <6.7 kb, or longer, i.e., ≥6.7 kb). The selection of age 35 as the tested cut-off point in this study was to mirror clinical practice, particularly in the United States, where NMDP focuses on recruiting donors aged 18–35 years. The donor age-LTL cumulative mortality risk analysis was not completed for mismatched or haplo-identical donors because of small numbers resulting in model non-convergence. The confidence intervals and p-values for cumulative risk estimates were calculated using a bootstrap with 1000 resamples.

We compared the two tested TL measurement methods (SB and qPCR) using Pearson correlation coefficient for LTL and LT/S on a continuous scale, and Kappa statistics to test for agreement of quartile categories assigned for LTL and LT/S. OS analyses for qPCR LT/S and LT/S-3M compared the lowest quartile to all others. Statistical analyses were performed using SAS 9.4 (SAS Institute; Cary, NC) and R version 4.3.0 (R Foundation for Statistical Computing); p ≤ 0.05 was considered statistically significant.

### Ethics

All study participants or their guardians provided informed consent for participation in the BMT CTN 1202 Protocol (NCT101879072) and the CIBMTR Research Database and Research Sample Repository Protocols (NCT01166009 and NCT00495300). The use of the clinical data and biospecimens for this study was approved by the Institutional Review Board of the NMDP and the BMT CTN Executive Committee.

### Role of funders

The study is funded by the NIH grant U01AG066529 (The Telomere Research Network), and by the intramural program of the National Cancer Institute, NIH. The Cancer Genomics Research Laboratory is funded with federal funds from the NCI, NIH, under NCI Contract 75N910D00024. The Centre for International Blood and Marrow Transplant Research (CIBMTR) is supported primarily by Public Health Service U24CA076518 from the NCI, the National Heart, Lung and Blood Institute and the National Institute of Allergy and Infectious Diseases; HHSH250201700006C from the Health Resources and Services Administration; and N00014-21-1-2954 and N00014-20-1-2832 from the Office of Naval Research. Support for this study was provided by grants U10HL069294 and U24HL138660 to the Blood and Marrow Transplant Clinical Trials Network (BMT CTN) from the National Heart, Lung, and Blood Institute and the National Cancer Institute. T.P.L work was supported by NSF grant 2032119, NIH grants 1U01AG066529, 3U01AG066529-02S1, NCI contract 75N91019P00829, and New Jersey Alliance for Clinical and Translational Science Career Development Award NJACTS KL2 TR003018. The content is solely the responsibility of the authors and does not necessarily represent the official views of the NIH and the above-mentioned parties. The manuscript was prepared using BMT CTN 1202 Research Materials obtained from the BMT CTN Repository operated by the NMDP and does not necessarily reflect the opinions or views of the BMT CTN 1202 protocol team, the BMT CTN, the NHLBI, or NCI.

## Results

### Patient characteristics

The study included 384 HCT recipients from 38 U.S. transplant centres. The recipient median age at HCT was 54.7 years (range = 3.2–76.3 years). The median age of the donors was 34.3 years (range = 2.3–76.2 years). Most patients had acute myeloid leukaemia (AML) or MDS (82.0%), the majority received peripheral blood stem cell grafts (73.4%), myeloablative regimens (63.3%), and methotrexate-based graft-vs-host disease prophylaxis (61.5%). Twenty-seven patients (7%) received post-transplant cyclophosphamide GVHD prophylaxis. Table 1 summarizes patient and transplant factors.

### LTL of donors pre-HCT and in recipients three months post-HCT

**Fig. 1:**
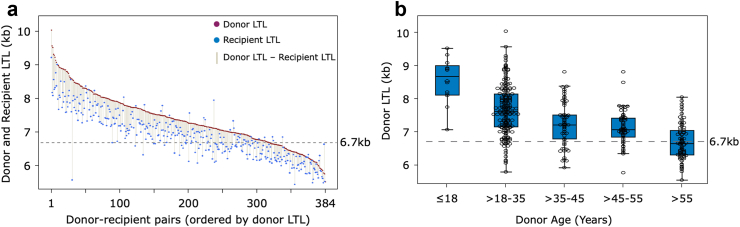
**Leucocyte telomere length (LTL), measured by Southern blotting in donors pre-haematopoietic cell transplant (HCT) and recipients at an average of 90 days post-HCT.** a). Magnitude of LTL-3MS (vertical grey lines) connecting LTLs of donors pre-HCT (red dots) and LTLs of respective HCT recipients (blue dots) at an average of 90 days post-HCT. Data are arranged along the x-axis (such that #1 designates the donor with the longest pre-HCT LTL and #384 designates the donor with the shortest LTL). There is considerable variation in the magnitude of telomere shortening 90 days post-HCT. This shortening, regardless of donors' LTL, is greater in HCT recipients from donors with longer LTL. b) Donor LTLs arranged by age groups. Interrupted horizontal lines in both panels denote the 6.7 kb threshold of donors' LTLs.

Fig. 1a displays the LTL of donors pre-HCT and that of their matched recipients three months post-HCT. Donor LTL ranged between 5.5 and 10 kb with a mean of 7.4 kb. The frequencies of LTL less than the 6.7 kb threshold by donor age were as follows: 0% in donors ≤18 years, 8.6% in donors >18–35 years, 22.8% in donors >35–45 years, 18.5% in donors >45–55 years, and 54.1% in donors >55 years (Fig. 1b). Recipient LTL post-HCT strongly correlated with the donor LTL before HCT (r = 0.89; p < 0.0001). Most patients (344/370; 93%) experienced LTL shortening three months after HCT with a mean of 444 bp (SD = 358 bp) but the magnitude of LTL-3MS was smaller for recipients of HCT from donors with LTL <6.7 kb than that of donors with LTL ≥6.7 kb (mean LTL-3MS = 263 bp vs 490 bp, respectively, p < 0.0001).

### Relationship between donor LTL or LTL-3MS with outcomes

**Fig. 2:**
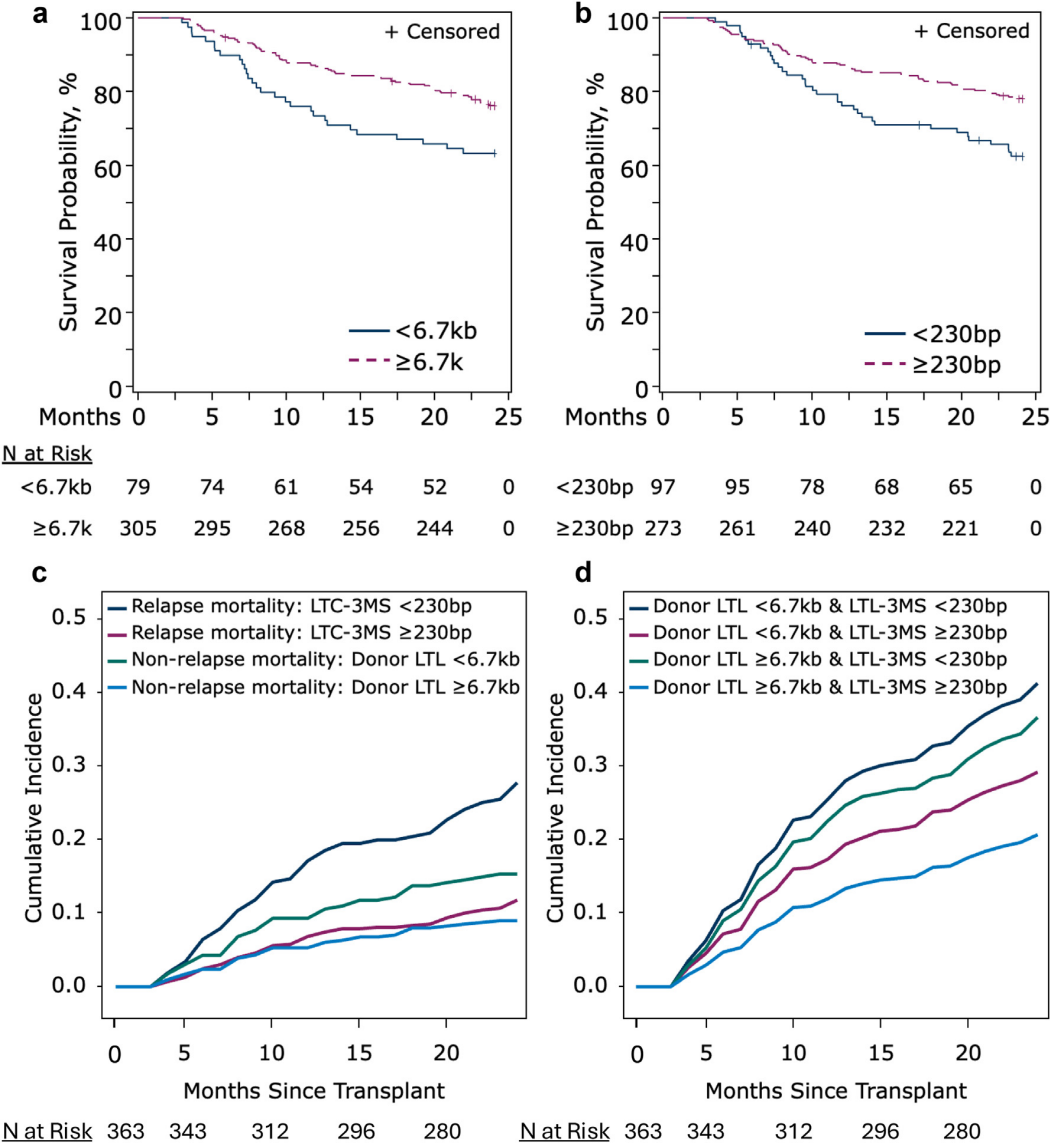
**Relations between two-year overall survival (OS) post-HCT and LTL parameters for early-stage leukaemia or myelodysplastic syndrome.** a) OS with donor LTL. b) OS with LTL shortening in recipients at three months post-HCT (LTL-3MS). c) Adjusted cumulative risks of cause-specific mortality with LTL parameters, separately for relapse mortality and non-relapse mortality. d) Combined effect of LTL parameters on the adjusted cumulative risk of all-cause mortality.

Two-year OS probability was higher for donor LTL ≥6.7 kb (76%) vs < 6.7 kb (63%) (p = 0.009). It was also higher for LTL-3MS ≥ 230 bp (78%) vs < 230 bp (62%) (p = 0.003) (Fig. 2a and b).

Multivariable analyses, adjusting for donor age and relevant clinical factors, confirmed the observed associations between each of the LTL parameters and OS (hazard ratio, HR with donor LTL ≥6.7 kb = 0.60, 95% CI = 0.37–0.96; p = 0.03; and HR with LTL-3MS ≥ 230 bp = 0.52, 95% CI = 0.34–0.80, p = 0.003; Table 2). Similar patterns of better OS with donor LTL ≥6.7 kb (p = 0.09) or LTL-3MS ≥ 230 bp (p = 0.003) were observed in analyses adjusted for centre, restricted on patients with AML, ALL, or MDS, or stratified by donor type (matched sibling, unrelated, and haplo-identical donors), or after removing donor age from the model (all with p < 0.05; Supplemental Tables S3 & S4). No interaction between donor LTL and LTL-3MS was noted in association with OS (p-interaction = 0.75).

**Table 2:** Multivariable associations between LTL parameters and HCT outcomes in HCT recipients for early-stage haematological malignancies.

	N event/Total	HR^a^ (95% CI)	p
**Donor LTL (≥6.7 kb vs < 6.7 kb)**			
All-cause mortality	100/383	0.60 (0.37–0.96)	0.03
Relapse Risk	85/360	0.89 (0.51–1.56)	0.69
Non-relapse mortality	37/360	0.48 (0.22–1.00)	0.05
**LTL-3MS (≥230 bp vs < 230 bp)**			
All-cause mortality	95/369	0.52 (0.34–0.80)	0.003
Relapse risk	78/347	0.53 (0.33–0.84)	0.008
Non-relapse mortality	37/347	0.96 (0.45–2.04)	0.91

Abbreviations: LTL, leucocyte telomere length; LTL-3MS: magnitude of telomere shortening three months after HCT.

a Models for all-cause mortality were adjusted for recipient and donor age, HCT-ci, sex, and disease type. Models for NRM were adjusted for recipient age, and HCT-ci. Models for relapse were adjusted for patient sex, HCT-ci, donor type.

L/TS showed no statistically significant associations between patient OS and donor LT/S (log rank p = 0.31) or LTL shortening in recipients at three months after HCT (LT/S-3MS; log rank p = 0.16; Supplemental Figure S2). These findings likely arise from the modest correlations between qPCR and SB measurements, and the misclassifications of TL/S particularly affecting the shortest quartile of TL (Supplemental Table S5 & Supplemental Figure S3).

We next evaluated the adjusted associations between LTL parameters and the two main causes of death in HCT recipients (NRM or disease relapse). Donor LTL ≥6.7 kb was associated with a lower risk of NRM than donor LTL <6.7 kb (HR = 0.48, 95% CI = 0.22–1.00, p = 0.05; and HR after adjusting for LTL-3MS = 0.35, 95% CI = 0.16–0.76, p = 0.008). Yet, there was no statistically significant association with relapse risk (HR = 0.89, p = 0.69) (Table 2). The corresponding adjusted two-year cumulative risk of NRM were 9% (95% CI = 6.2–12.5%) and 15% (95% CI = 7.5–23.2%) in HCT recipients from donors with LTL ≥6.7 kb and <6.7 kb, respectively (p = 0.17). LTL-3MS ≥ 230 vs < 230 bp was associated with lower risk of relapse (HR = 0.53, 95% CI = 0.33–0.84, p = 0.008) with no association with NRM (HR = 0.96, p = 0.91) (Fig. 2C). The 2-year cumulative risks of relapse mortality were 12% (95% CI = 10.9–12.7%) and 28% (95% CI = 25.0–30.6%) in HCT recipients with LTL-3MS of ≥230 bp and <230 bp, respectively, p < 0.0001 (Fig. 2C). The association between LTL-3MS and relapse risk remained significant (HR = 0.51, 95% CI = 0.31–0.82, p = 0.006) after adding donor LTL and intensity of conditioning regimen to the model. Additionally, no statistically significant interaction between conditioning regimen intensity and LTL-3MS with relapse risk was noted (HR = 0.38 and 0.68 in myeloablative, and reduced intensity/non-myeloablative regimens, respectively; p-interaction = 0.30).

When considering the combined effect of LTL parameters on all cause-mortality, the cumulative risk was almost reduced by half for patients with both donor LTL ≥6.7 kb and LTL-3MS ≥ 230 bp, as compared to patients with neither characteristic (2-year adjusted cumulative risk of death = 21%, 95% CI = 17.8–24.2% vs 41%, 95% CI = 34.0–48.2, respectively; p < 0.0001) (Fig. 2D).

### Donor LTL vs age for donor selection criteria

Because donor age is an established risk factor for patient survival after HCT, we examined its effect in this study (Supplemental Table S6). The detected OS associations showed the expected survival improvement with younger donors, though given the relatively small sample size, the association did not reach statistical significance. The HR with donor age of ≤ vs >35 years was 0.80 (95% CI 0.53–1.20).

**Fig. 3:**
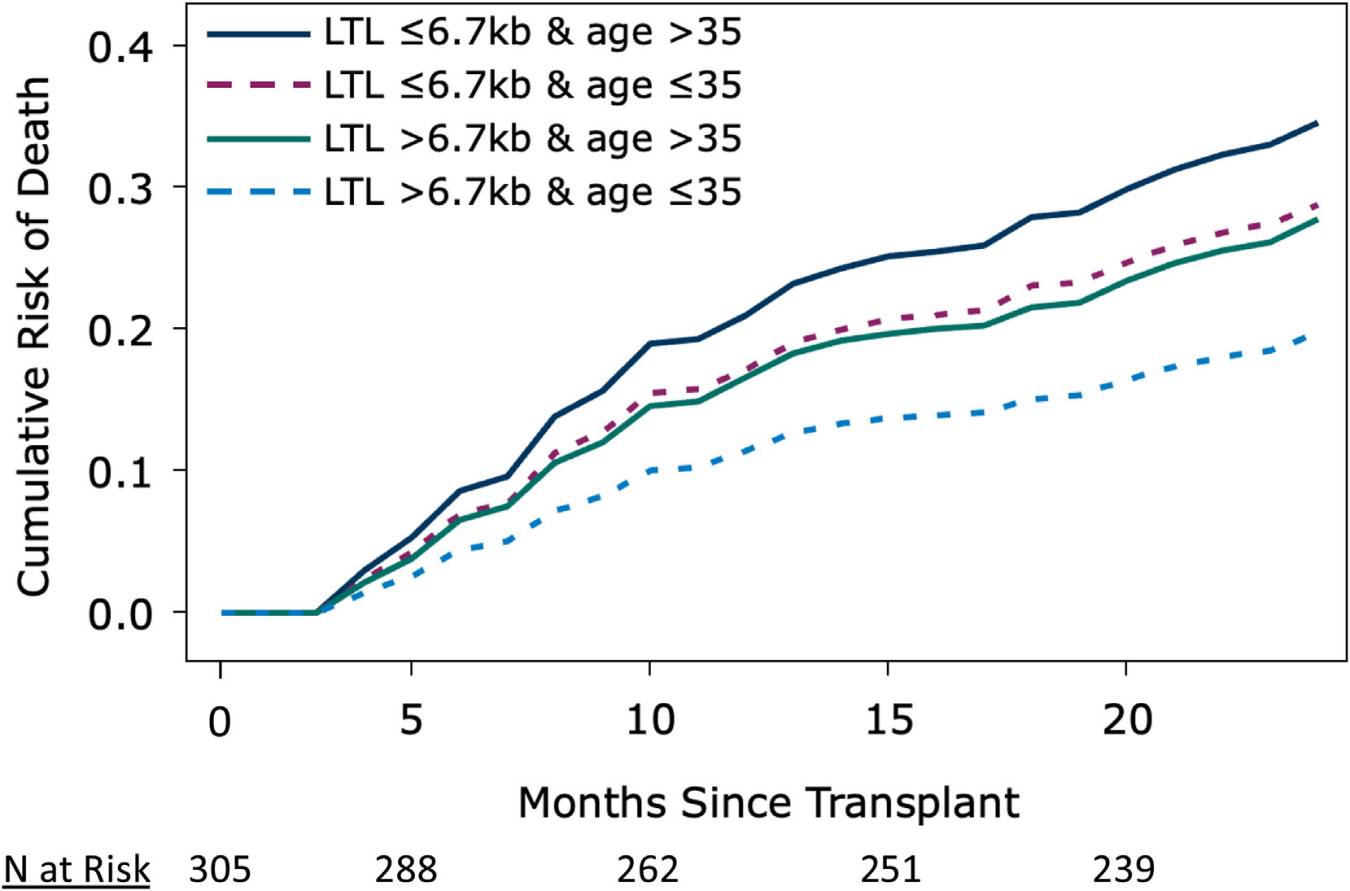
**Adjusted cumulative risk of death by donor LTL and age in matched donor transplants for early-stage leukaemia or myelodysplastic syndrome**.

We next examined the effect of LTL as a new donor selection criterion by calculating the 2-year adjusted cumulative risk of all-cause mortality by strata of donor age (age ≤35 vs age >35 years) and donor LTL (<6.7 kb vs ≥ 6.7 kb) among HLA-matched transplants. The cumulative risk of two-year mortality was significantly lower when donors were younger (age ≤35 years) with longer LTL (≥6.7 kb) vs older age (>35 years) with shorter LTL (<6.7 kb) (cumulative risk = 19.7% vs 34.6%, p = 0.008). Longer LTL appeared to provide survival benefits in both younger and older donor groups (2-year cumulative risk of mortality = 19.7% vs 28.8%, p = 0.09, for donor LTL ≥6.7 kb vs <6.7 kb among younger donors; and the cumulative risk = 27.7% vs 34.6%, p = 0.15, respectively among older donors) (Fig. 3).

## Discussion

HCT is a curative treatment for haematological malignancies, but its risks of morbidity and mortality remain high.^16^^,^^17^ Our study found a higher OS in patients receiving HCT for early-stage leukaemia and MDS from donors with longer LTL than from donors with shorter LTL. This relationship was independent of donor age. The study also uncovered an association of lower risk of disease relapse with higher post-HCT LTL shortening. These findings underscore the likely crucial role of LTL-dependent replicative capacity of donated HCs in HCT outcomes. This is especially important given the extensive data linking younger donor age with better HCT outcomes,^3^^,^^4^^,^^18^^,^^19^ and the current preference of donor registries to recruit young donors (18–35 years of age).

HC telomeres shorten with age due to HC replication throughout an individual's lifespan. Consequently, older persons typically have shorter HC telomeres than younger ones. However, age is a weak surrogate for LTL and wide variation exists across individuals of the same age (LTL SD is about 700 bp in the general population and the current study).^7^^,^^20^ Donor LTL showed a modest negative correlation with age in the current study (r = −0.54, Supplemental Figure S4). Therefore, some young donors have LTL <6.7 kb threshold, while many older donors have LTL ≥6.7 kb. Indeed, in our study, one out of 12 donors younger than 35 years had LTL <6.7 kb, while more than half of donors older than 35 years had LTL ≥6.7 kb. If donor LTL is a key factor in the improved outcomes of HCT from younger donors, then direct measurement of donor LTL could improve donor selection by excluding younger donors with shorter LTL and including older donors with longer LTL. This approach will not only improve HCT outcomes but also vastly expand donor eligibility.

Recipients of HCs from donors with LTL <6.7 kb had a lower OS primarily because of NRM, perhaps because of incomplete reconstitution of the haematopoietic and immune systems, stemming from diminished TL-dependent replicative capacity of donated HCs with shorter telomeres. A previous study in patients receiving HCT for severe aplastic anaemia (SAA) found that longer donor LTL was associated with a lower risk of infection-related death.^21^

Donated HCs experience multiple replications after HCT, leading to telomere shortening, as shown in LTL-3MS (average shortening = 444 bp). To provide context, this level of shortening is roughly equivalent to LTL shortening over 15 years in healthy adults.^20^ One intriguing finding is the reduced risk of relapse that we observed in recipients with LTL-3MS ≥230 bp. Since the shortening of telomeres in transplanted HCs also stems from the extra-medullary expansion of T cells,^22^ we speculate that a higher LTL-3MS signifies a more robust expansion of the T cell compartment after HCT. This enhanced T-cell expansion may ultimately contribute to better outcomes in terms of “relapse-free survival”.^23^ Measurements of telomere length in leucocyte subsets, T-cells, in particular, would generate a deeper understanding of the role of telomere length dynamics post-HCT in HCT outcomes.

We note that the threshold effect we observed in donor LTL and LTL-3MS follows principles of TL dynamics. While the proliferation of transplanted HCs in recipients post-HCT follows an essentially exponential pattern, the shortening of telomeres in these cells progresses linearly.^24^ This means that the expansions of donated haematopoietic cells drop by 50% with each TL-dependent replicative cycle missed, which can occur as telomeres become too short, limiting further replication. For example, if the replicative capacity of a donated haematopoietic cell decreases from 20 to 15 replications, the maximum size of its expansion drops from approximately one million to 30 thousand cells. Therefore, the impact of telomere length on donated haematopoietic cells manifests mainly when certain thresholds are reached.

Our findings add to the evidence supporting a role for donor LTL in post-HCT survival, as previously shown in patients with SAA.^21^^,^^25^^,^^26^ However, these findings challenge previous research that found no effect of donor LTL, measured by qPCR, on outcomes of HCT recipients for haematopoietic neoplasms.^27^^,^^28^ The inability of our LT/S data to capture associations of TL parameters with HCT outcomes highlights the known limitations of the qPCR method.^29^^,^^30^ A recent study comparing several qPCR LT/S protocols with SB LTL showed a wide variation in the precision of qPCR protocols with an intraclass coefficient (ICC) ranging between 0.43 and 0.94 as compared to an ICC of 0.99 for SB.^31^ We note that using qPCR, a previous study in nonmyeloablative HCT for haematological malignancies showed an association between high post-HCT LTL shortening and increased risk of NRM.^28^ This finding does not necessarily conflict with our findings since it relates to relapse-free survivors with LTL shortening at 9–15 months post-HCT.

We did not measure recipient LTL pre-HCT, which might have an impact on patient outcomes. Short recipient LTL (measured by qPCR) in patients with SAA or MDS, but not in acute leukaemia, was associated with a higher risk of transplant-related mortality.^32^, ^33^, ^34^ The possible interaction between LTL in recipients and donors pre-HCT has not been well studied. Still, results from an earlier SAA study suggested that the survival advantage associated with longer donor LTL was independent of recipient LTL.^25^

We acknowledge the study limitations, including a relatively brief follow-up period of two years. The study included patients with early-stage AML and MDS, and therefore the results might not be generalizable to other patients. Additionally, the current study did not include patients who died in the first three months after HCT, which can result in selection bias. However, the relatively low frequency of death during this period and its overwhelming majority of NRM assure the validity of our findings.

We were not able to account for the possibility of mixed donor chimaerism in patients with non-myeloablative or reduced intensity conditioning regimens because of lacking information. However, we found no statistically significant difference between the relative risk of relapse in relation to LTL-3MS by conditioning regimen intensity. The study lacked information on prognostic karyotypes or molecular genetic testing. Such information may explain some of the study findings.

In conclusion, we found that longer donor pre-HCT LTL and a higher rate of LTL shortening post-HCT are associated with better outcomes for early-stage leukaemia or MDS. These findings, if validated, have the potential to change donor selection strategies for allogeneic HCT. The LTL parameter thresholds used in this study should be considered as a general guideline and need to be refined in future studies to allow for a systematic threshold selection of donor LTL in clinical settings.

## Contributors

Study design and Funding: SMG and AA.

Laboratory work and telomere measurements: AA, TPL, CLD, and AAH.

Data access and management: CP, VS, WS, and SS.

Statistical analysis and support: SMG, HK, PA, and JA.

Data interpretation: All.

Manuscript draft: SMG and AA.

Critical review: All.

All authors read and approved the final version of the manuscript.

## Data sharing statement

Deidentified data from this study are available upon request (email: gadallas@nih.gov). Data access permission will require a material transfer agreement.

## Declaration of interests

No conflict of interests to declare.

## Appendix A Supplementary data

Supplemental Material Supplementary Table S1. Relationship between donor LTL parameters on a continuous scale and overall survival of recipients post-HCT. Supplementary Table S2: Table S2: Distributions of LTL parameters in donors pre-HCT and recipients after HCT (LTL-3MS). Supplementary Table S3: Associations between LTL parameters and overall survival of HCT recipients for early-stage haematological malignancies. Supplementary Table S4: Relationship between donor LTL parameters and overall survival of HCT recipients for early-stage haematological malignancies. Supplementary Table S5: Agreement between Southern blotting and qPCR telomere length measurements. Supplementary Table S6: The associations between donor age (≤35 years *vs.* >35 years) and HCT outcomes. Supplementary Figure S1: Probabilities of overall survival in early-stage haematological malignancies and quartile categories of donor LTL parameters. A) Pre-HCT donor LTL; B) Magnitude of LTL shortening at three-month post-HCT (LTL-3MS). Supplementary Figure S2: Post-HCT survival probabilities in HCT recipients for early-stage haematological malignant at HCT A) by donor qPCR telomere length (qPCR TL; Q1 is the shortest quartile); B) by qPCR TL magnitude of post-HCT shortening (Q1 is the least quartile of TL shortening). Supplementary Figure S3: The correlations between telomere length measurement methods. A) for donor telomere length (LTL is southern blotting measurements and LT/S is qPCR measurements; B) for post-HCT telomere length shortening in recipients at 3 months (LTL-3M is southern blotting measurements and LTS-3M is qPCR measurements). Supplementary Figure S4: The correlation between donor age and LTL.
