# Supplementary material for: Donor telomeres and their magnitude of shortening post-allogeneic haematopoietic cell transplant impact survival for patients with early-stage leukaemia or myelodysplastic syndrome
Source: eBioMedicine. 2025 Mar 8;114:105641. doi: 10.1016/j.ebiom.2025.105641 (PMC11930427; doi:10.1016/j.ebiom.2025.105641)

**Table S1**

| **Donor LTL** | |
| --- | --- |
| HR^a^ (95% CI) | *p* |
| 0.86 (0.64-1.17) | 0.34 |
| **LTL-3MS** | |
| HR* (95% CI) | *p* |
| 0.67 (0.37-1.24) | 0.20 |

^a^Models were adjusted for recipient and donor age, HCT-ci, sex, and disease type.

**Table S2**

|  | Mean (SD) |
| --- | --- |
| Donor LTL (kb) | 7.37 (0.79) |
| Post-HCT LTL (kb) | 6.92 (0.70) |
| LTL-3MS (bp) | 440 (360) |
| LTL-3MS by donor LTL (bp) |  |
| Donors with LTL < 6.7 kb | 260 (270) |
| Donors with LTL ≥ 6.7 kb | 490 (360) |
|  | N (%) |
| Donor TL |  |
| < 6.7 kb | 79 (20.57%) |
| ≥ 6.7 kb | 305 (79.43%) |
| LTL-3MS |  |
| < 230 bp | 97 (26.22%) |
| ≥ 230 bp | 273 (73.78%) |

Total N=384 (all have donor LTL); recipient LTL post-HCT was missing in 14 patients Abbreviations: LTL: leukocyte telomere length; LTL-3MS, LTL shortening in recipients at three months post-HCT.

**Table S3**

|  | *Donor-age unadjusted* | *Adjusted for centres* | *Restricted on AML, ALL, and MDS* |
| --- | --- | --- | --- |
|  | *HR^a^ (95% CI)*  *P* | *HR^b^ (95% CI)*  *P* | *HR^c^ (95% CI)*  *P* |
| **Donor LTL**  **(≥6.7kb *vs.* < 6.7 kb)** | 0.59 (0.38-0.93)  0.02 | 0.66 (0.40-1.08)  0.09 | 0.60 (0.38-0.95)  0.03 |
| **LTL-3MS**  **(≥230 bp *vs.* < 230 bp)** | 0.52 (0.34-0.79)  0.002 | 0.52 (0.34-0.81)  0.003 | 0.55 (0.36-0.85)  0.007 |

Abbreviations: LTL, leukocyte telomere length; LTL-3MS: magnitude of telomere shortening three months after HCT

1. Adjusted for recipient age, HCT-ci, sex, and disease type.
2. Adjusted for recipient and donor ge, HCT-ci, sex, disease type, and centre.
3. Adjusted for recipient and donor ge, HCT-ci, sex, and disease type.

**Table S4**

| **Donor LTL (≥6.7kb *vs.* < 6.7 kb)** | | |
| --- | --- | --- |
| Unrelated | Matched siblings | Haplo-identical |
| HR^a^ (95% CI); p | | |
| 0.55 (0.27-1.12); p=0.10 | 0.47 (0.22-0.99); p=0.05 | 0.61 (0.04-8.61; p=0.72 |
| **LTL-3MS (≥230 bp *vs.* < 230 bp)** | | |
| Unrelated | Matched siblings | Haplo-identical |
| HR^a^ (95% CI); p | | |
| 0.51 (0.26-0.96); p=0.04 | 0.44 (0.22-0.88); p=0.02 | 0.38 (0.08-1.82); p=0.38 |

^a^ Models were adjusted for recipient and donor age, HCT-ci, sex, and disease type.

**Table S5**

|  | Donor Telomere Length | | Post-HCT telomere Shortening | |
| --- | --- | --- | --- | --- |
|  | qPCR LT/S (Q1) | qPCR LT/S  (Q2-Q4) | qPCR LT/S-3MS (Q1) | qPCR LTS-3MS (Q2-Q4) |
|  |  | N (%)^a^ |  |  |
| SB LTL/LTL-3MS  (Q1) | 41 (52.6%) | 37 (47.4%) | 25 (25.8%) | 72 (74.2%) |
| SB LTL (Q2-Q4) | 53 (17.5%) | 249 (82.5%) | 67 (24.5%) | 206 (75.5%) |
| Kappa coefficient (95% CI) | 0.33 (0.21-0.43) | | 0.01 (-0.09-0.11) | |

^a^row percent; N with donor telomere length with both assays=380; N with calculated telomere shortening with both assays=370

**Table S6**

|  | Without donor LTL adjustment | | With donor LTL adjustment | |
| --- | --- | --- | --- | --- |
|  | HR (95% CI) | P | HR (95% CI) | P |
| All-cause mortality | 0.80 (0.53-1.20) | 0.28 | 0.88 (0.57-1.33) | 0.53 |
| Relapse Risk | 0.88 (0.55-1.42) | 0.60 | 0. 90 (0.55-1.56) | 0.66 |
| Non-relapse Mortality | 0.82 (0.42-1.57) | 0.54 | 0.94 (0.47-1.86) | 0.86 |

All-cause mortality model was adjusted for recipient age, HCT-ci, sex, and disease type; relapse model was adjusted for patient sex, HCT-ci, donor type; and NRM model was adjusted for recipient age, and HCT-ci.

**Figure S1**

| 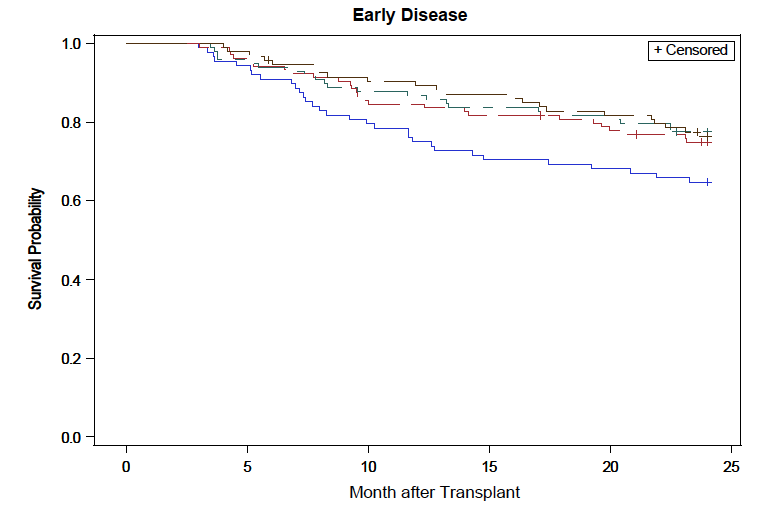  A)    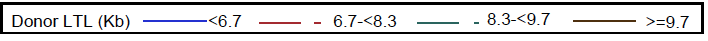 |
| --- |
| **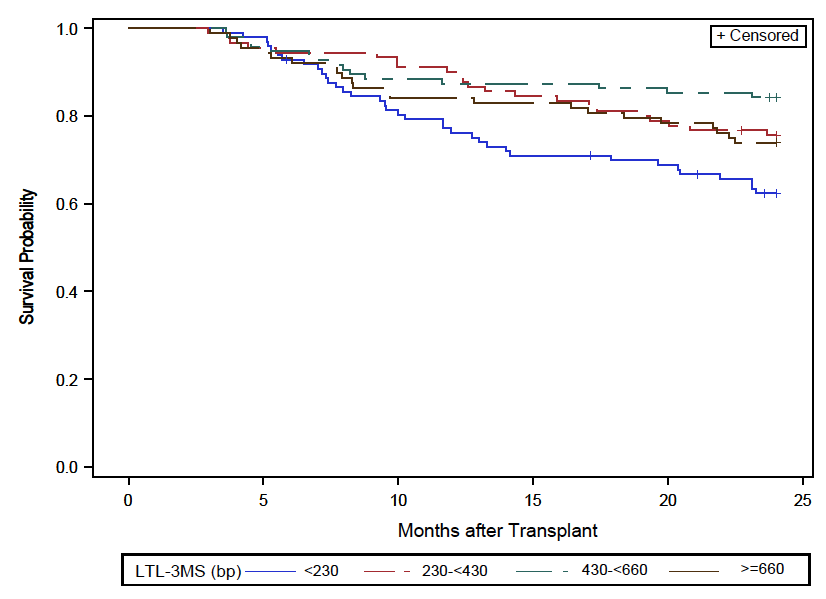**  B) |

**Figure S2**

| A) | 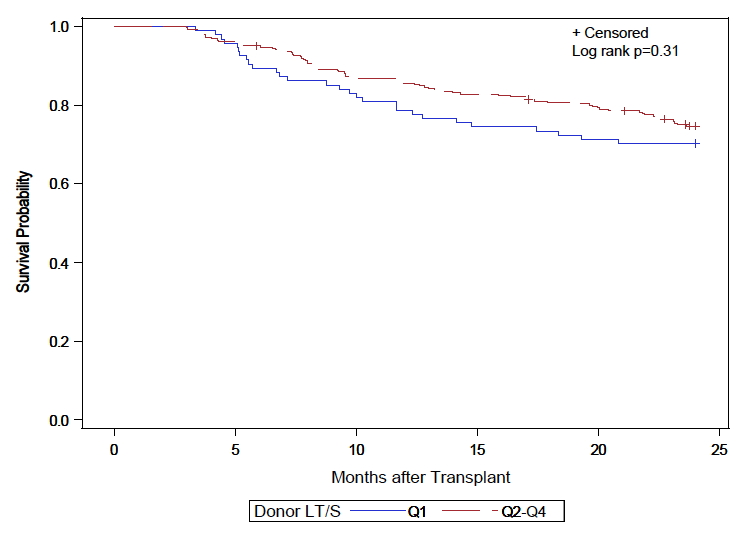 |
| --- | --- |
| B) | 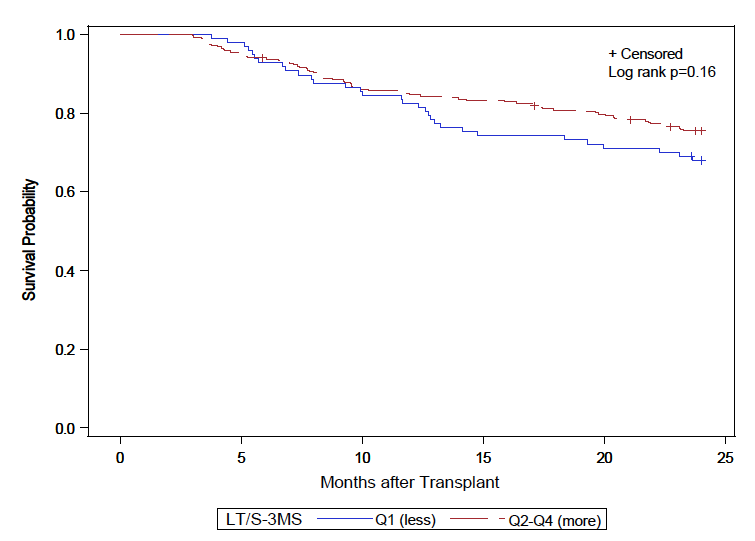 |

**Figure S3**

A)


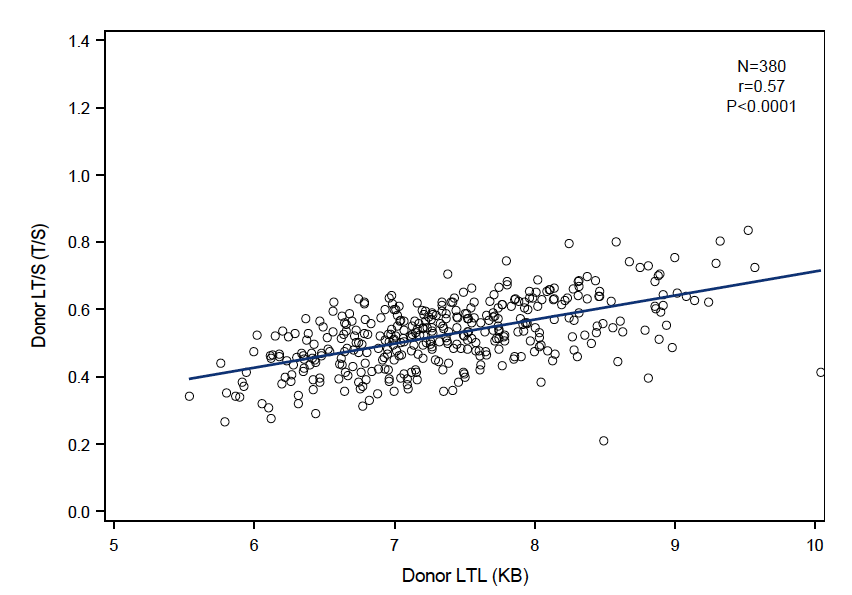


B)


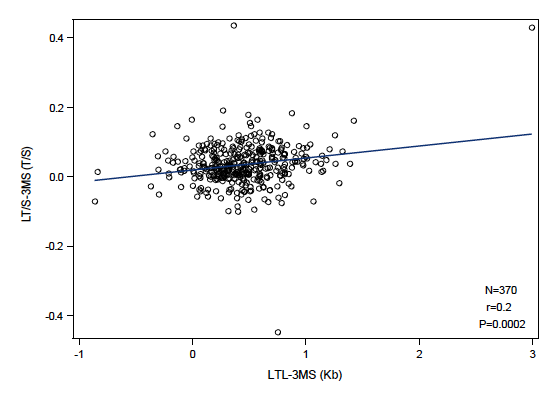


**Figure S4**


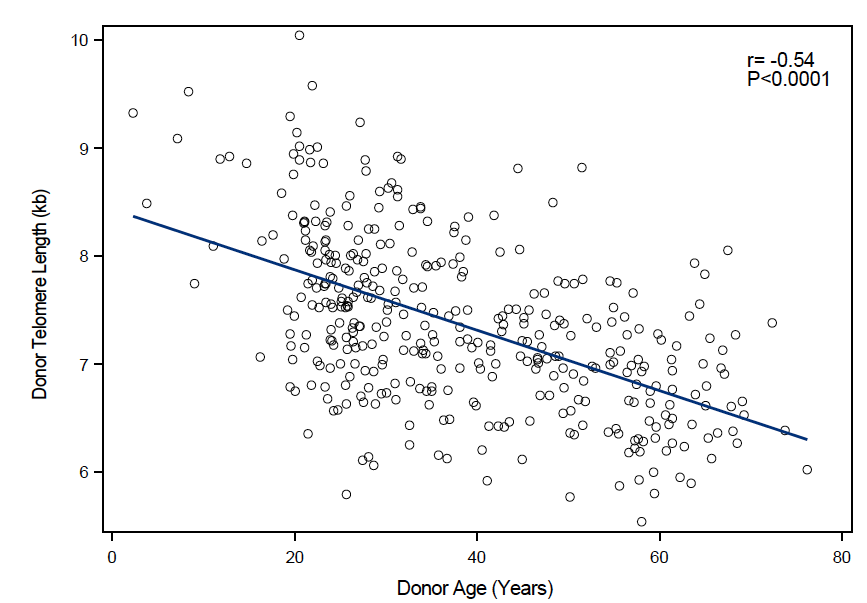

Supplement: Supplemental Material — Supplementary Table S1. Relationship between donor LTL parameters on a continuous scale and overall survival of recipients post-HCT. Supplementary Table S2: Table S2: Distributions of LTL parameters in donors pre-HCT and recipients after HCT (LTL-3MS). Supplementary Table S3: Associations between LTL parameters and overall survival of HCT recipients for early-stage haematological malignancies. Supplementary Table S4: Relationship between donor LTL parameters and overall survival of HCT recipients for early-stage haematological malignancies. Supplementary Table S5: Agreement between Southern blotting and qPCR telomere length measurements. Supplementary Table S6: The associations between donor age (≤35 years vs. >35 years) and HCT outcomes. Supplementary Figure S1: Probabilities of overall survival in early-stage haematological malignancies and quartile categories of donor LTL parameters. A) Pre-HCT donor LTL; B) Magnitude of LTL shortening at three-month post-HCT (LTL-3MS). Supplementary Figure S2: Post-HCT survival probabilities in HCT recipients for early-stage haematological malignant at HCT A) by donor qPCR telomere length (qPCR TL; Q1 is the shortest quartile); B) by qPCR TL magnitude of post-HCT shortening (Q1 is the least quartile of TL shortening). Supplementary Figure S3: The correlations between telomere length measurement methods. A) for donor telomere length (LTL is southern blotting measurements and LT/S is qPCR measurements; B) for post-HCT telomere length shortening in recipients at 3 months (LTL-3M is southern blotting measurements and LTS-3M is qPCR measurements). Supplementary Figure S4: The correlation between donor age and LTL. [file mmc1.docx]
